# Prothrombin Complex Concentrate for Warfarin-Associated Intracranial Bleeding in Neurosurgical Patients: A Single-Center Experience

**DOI:** 10.3390/medicina54020022

**Published:** 2018-04-25

**Authors:** Jomantė Mačiukaitienė, Diana Bilskienė, Arimantas Tamašauskas, Adomas Bunevičius

**Affiliations:** 1 Faculty of Medicine, Medical Academy, Lithuanian University of Health Sciences, LT-50009 Kaunas, Lithuania; j.maciukaitiene@gmail.com; 2 Department of Anesthesiology, Medical Academy, Lithuanian University of Health Sciences, LT-50009 Kaunas, Lithuania; diana.bilskiene@kaunoklinikos.lt; 3 Neuroscience Institute, Medical Academy, Lithuanian University of Health Sciences, LT-50009 Kaunas, Lithuania; arimantas.tamasauskas@lsmuni.lt; 4 Department of Neurosurgery, Medical Academy, Lithuanian University of Health Sciences, LT-50009 Kaunas, Lithuania

**Keywords:** intracranial hemorrhage, warfarin, prothrombin complex concentrate

## Abstract

*Objective*: The number of patients presenting with warfarin-associated intracranial bleeding and needing neurosurgical intervention is growing. Prothrombin complex concentrate (PCC) is commonly used for anti-coagulation reversal before emergent surgery. We present our experience with PCC use in patients presenting with coagulopathy and needing urgent craniotomy. *Methods*: We retrospectively identified all patients presenting with intracranial bleeding and coagulopathy due to warfarin use, requiring urgent neurosurgical procedures, from January, 2014 (implementation of 4-PCC therapy) until December, 2016. For coagulation reversal, all patients received 4-PCC (Octaplex) and vitamin K. *Results*: Thirty-five consecutive patients (17 men; median age 72 years) were administered 4-PCC before emergent neurosurgical procedures. The majority of patients presented with traumatic subdural hematoma (62%) and spontaneous intracerebral hemorrhage (32%). All patients were taking warfarin. Median international normalized ratio (INR) on admission was 2.94 (range: 1.20 to 8.60). Median 4-PCC dose was 2000 I.U. (range: 500 I.U. to 3000 I.U.). There was a statically significant decrease in INR (*p* < 0.01), PT (*p* < 0.01), and PTT (*p* = 0.02) after 4-PCC administration. Postoperative INR values were ≤3.00 in all patients, and seven (20%) patients had normal INR values. There were no 4-PCC related complications. Four (11%) patients developed subdural/epidural hematoma and 20 (57%) patients died. Mortality was associated with lower Glasgow coma scale (GCS) score. *Conclusions*: The 4-PCC facilitates INR reversal and surgery in patients presenting with warfarin-associated coagulopathy and intracranial bleeding requiring urgent neurosurgical intervention.

## 1. Introduction

With increasing life expectancy, the number of patients needing long-term anticoagulation is constantly growing [1]. Warfarin remains the most commonly used drug for long-term anticoagulation [2]. It is a vitamin K antagonist (VKA) that inhibits synthesis of clotting factors II, VII, IX, and X in the liver. Due to narrow therapeutic index, patients taking warfarin require careful monitoring and frequent dose adjustments in order to maintain therapeutic concentration and to prevent warfarin toxicity [3]. Nevertheless, warfarin overdose is common, and is often associated with hemorrhagic complications, including intracranial bleeding [1]. Larger hematoma volume and worse level of consciousness in patients presenting with warfarin-associated intracranial bleeding are independently associated with poor discharge and long-term functional outcomes [4].

In patients presenting with intracranial bleeding, prompt reversal of anticoagulation is important in order to decreases the risk of intracranial bleeding expansion [5]. Currently available VKA reversal options include vitamin K, fresh frozen plasma (FFP), prothrombin complex concentrate (PCC), and recombinant factor VII [1]. PCC is recommended as the first-line agent for warfarin reversal in emergency situations [2]. PCC is preferred over FFP because it does not require blood typing and cross-matching, and eliminates the risk of FFP-related volume overload. Furthermore, normalization of the international normalized ratio (INR) is significantly faster after administration of PCC than FFP [5]. For example, a study in patients presenting with anticoagulation-associated intracerebral hemorrhage documented that addition of PCC to FFP and vitamin K corrected INR in 80% of subjects, compared to 33% of patients who received Vitamin K and FFP [6]. PCC can include three clotting factors (II, IX, and X; 3-PCC) or four clotting factors (II, IX, X, and VII; 4-PCC) [7]. A recent meta-analysis indicated that 4-PCC provides faster and more stable INR normalization in patients taking warfarin than 3-PCC [7]. However, other authors did not find significant differences between 4-PCC and 3-PCC in the rate of INR reversal and clinical outcomes in patients requiring emergent warfarin reversal [8,9,10].

Neurosurgical procedures are associated with high bleeding risk; therefore, prompt and reliable reversal of anticoagulation is critical in order to allow surgeons to perform emergent surgery, and to reduce the risk of intraoperative and postoperative bleeding complications. To date there have been a few attempts to study safety and efficacy of PCC in the neurosurgical setting; however, available studies were performed in the framework of clinical trials in mixed population of trauma patients [11], were focused on non-surgical intracerebral hemorrhage patients [12], or studied neurosurgical patients who required spinal procedures [13]. Studies exploring clinical utility of PCC in a routine neurosurgical setting, among patients requiring urgent intracranial neurosurgical intervention, remain sparse [14].

The goal of the present report is to present our experience regarding safety and efficacy of 4-PCC in consecutive patients presenting with warfarin-associated coagulopathy and requiring emergent neurosurgical procedures for intracranial bleeding.

## 2. Materials and Methods

The study was approved by Kaunas Regional Biomedical Research Ethics Committee, Kaunas, Lithuania (No. BE-2-17; issued on 21 June 2017). We retrospectively identified all patients admitted at the Department of Neurosurgery of the Hospital of Lithuanian University of Health Sciences, Kaunas, Lithuania, with acute intracranial bleeding who received anticoagulation reversal with 4-PCC since the implementation of anticoagulation reversal protocol with 4-PCC in January 2014, until December 2016.

Medical records were reviewed for demographics characteristics, admission diagnosis, clinical disease severity (Glasgow coma scale [GCS] [15] score and American society of anesthesiologists [ASA] physical status classification system [16]), past medical histories, and medication use. Routine pre-operative laboratory test results and coagulation panel before and after neurosurgical intervention were also recorded. Coagulation panel included INR (normal range: 0.9–1.2), prothrombin time (PT; normal range: 70–130%) and partial thromboplastin time (PTT; normal range: 28–38 s). We also recorded neurosurgical procedures and complications during the hospitalization period and outcome at hospital discharge. Postoperative non-contrast head CT was performed the day after the surgery in all patients. Surgical clot removal was performed as indicated by the treating physician and without the control of coagulation parameters, in order to save time and perform surgery as fast as possible. Outcome at discharge was evaluated using the Glasgow outcome scale (GOS) [17] that is validated and commonly used in Lithuania for research purposes [18].

Octaplex® (Octapharma, Langenfeld, Germany) is a 4-PCC that was used for anticoagulation reversal in all cases. The dose of Octaplex solution (500 I.U./20 mL saline) was selected according to the manufacturer’s instructions based on INR values and body weight [19]: Doses ranged from 0.9 to 1.2 mL/kg for patients with INR of <2.5; from 1.3 to 1.6 mL/kg for patients with INR of 2.5 to 3.0; from 1.7 to 1.9 mL/kg for patients with INR of 3.1 to 3.5; and >1.9 mL/kg for patients with INR of >3.5. The maximum dose of Octaplex® was 3000 I.U. 4-PCC dose was discussed with hematologists in all cases, as required by hospital/national regulations. The target INR level was ≤1.2. Intravenous infusion of 4-PCC was initiated before surgery and was completed during the neurosurgical procedure. All patients received 10 mg of intravenous vitamin K solution, which was initiated before 4-PCC infusion. Subsequent vitamin K administration was selected according to patient clinical status and PTT. Repeated 4-PCC dose was not administered, with the exception of patients requiring repeated neurosurgical intervention.

### Statistical Analysis

Statistical analysis was performed using the statistical package for the social sciences (Windows Version 23; SPSS). Distribution of continuous variables was evaluated using the Kolmogorov-Smirnov test. All continuous data were not normally distributed and are presented as median (range). Categorical data is presented as number (percent). Statistical comparisons were performed using the Mann-Whitney, Wilcoxon, and Pearson *X*^2^ tests. A *p*-value of <0.05 was selected as statistically significant.

## 3. Results

During the study period, 35 patients (17 men and 18 women; median age 72 years) received anticoagulation reversal with 4-PCC and underwent emergent surgery for intracranial bleeding (Table 1). Coagulopathy was attributed to warfarin use in all cases, and one patient also had documented liver cirrhosis. The majority of patients were operated on for traumatic acute subdural hematoma (53%), followed by supratentorial (20%) and infratentorial (12%) spontaneous intracerebral hemorrhage (ICH), and chronic subdural hematoma (9%). Two patients were operated upon for ruptured intracranial aneurism and bleeding into metastatic brain tumor. Median admission GCS score was 7. Two (6%) patients were also diagnosed with thrombocytopenia (platelet < 100 × 10^9^/L). The majority of patients were operated via craniotomy (66%).

Preoprative and postoperative INR, PT, and PTT values are presented in Table 2. Median INR on admission was 2.94, and 12 (35%) patients had severe coagulopathy, with INR values of >3.50. The 4-PCC infusion was initiated immediately after the decision was made to operate on the patient, and was completed in the operating room. Median 4-PCC dose was 2000 I.U. (range: 500–3000 I.U.). There were no side effects or complications attributed to 4-PCC infusion. All patients were also administered vitamin K. Platelet transfusion was performed for one patient, and fresh frozen plasma was administered to another patient. Four (11%) patients received red blood cell transfusion. Coagulation function improved after 4-PCC administration in all patients (Table 2). There was a statically significant decrease in INR (*p* < 0.01), PT (*p* < 0.01), and PTT (*p* = 0.02) after 4-PCC administration. Postoperative INR values were ≤3.00 in all patients, and 7 (20%) patients had INR values <1.20.

Wilcoxon and Pearson χ^2^ tests were used for continuous and categorical data, respectively. 

In-hospital complications occurred in 15 (43%) patients. All complications were diagnosed in patients who survived at least 5 days after the surgery (15/28; 54%). Post-operative intracranial bleeding that required repeated neurosurgical intervention was diagnosed in 4 (11%) patients: Two patients were re-operated for subdural hematoma recurrence, and two patients for new epidural hematoma (Table 3). Age, disease severity, INR values, platelet count, and 4-PCC dose were similar between patients, with or without postoperative intracranial bleeding (all *p*-values >0.20).

Median hospitalization duration was 13 days, and ranged from 1 day to 87 days in the total sample.

Thirty-one percent of patients had a favorable discharge outcome, and sixty-nine percent of patients had an unfavorable outcome. Twenty patients (57%) died in the hospital. Deaths were attributed to poor clinical status (60%) and infectious complications (40%). All deaths occurred in an intensive care unit, and there were no deaths in operating room. Patients who died presented with a lower GCS score than patients who survived (*p* = 0.06; Table 4). As expected, hospital stay was shorter among patients who died, relative to patients who survived. Patient age, INR values, platelet count, 4-PCC dose, and postoperative complication rates were similar between patients who died and survived (*p*-values > 0.13).

## 4. Discussion

In our experience, the 4-PCC was a safe and effective treatment method for reversal of warfarin-associated coagulopathy in patients requiring urgent neurosurgical intervention. Post-operative intracranial bleeding was diagnosed in 11% patients. Nevertheless, the mortality rate was high, and outcomes were poor, in patients with warfarin-associated coagulopathy, and were mainly attributed to greater disease severity. Our findings contribute to the growing literature showing that the PCC facilitates neurosurgical intervention in patients taking VKAs and requiring urgent neurosurgical intervention, and is associated with acceptable safety and efficacy [20,21,22,23,24,25,26,27,28,29,30,31,32].

The 4-PCC administration was safe and well-tolerated in the emergent neurosurgery setting, and there were no serious adverse events. The previously reported incidence rate of serious adverse events of 4-PCC ranged from 0.6 to 9.7%, and included ischemic stroke, myocardial infarction, heart failure, pulmonary embolism, deep venous thrombosis, and arterial thromboembolism [28]. In our cohort, patient mortality was attributed to severe clinical status in the majority of cases; however, pathological examination was not performed, or data is not available, therefore we cannot discern if thromboembolic complications were causes of death. High rate of infectious complication can be attributed to severe patient clinical status requiring prolonged mechanical ventilation, resulting in increased risk of pneumonia.

The 4-PCC dose was chosen on a case-by-case basis according to initial INR values, and ranged from 500 I.U. to 3000 I.U. All patients also received vitamin K, and platelets were transfused in patients with thrombocytopenia. There are various PCC dosing options, including fixed dosing, and dosing based on INR and/or weight [7]. The optimal dosing and/or maintenance schedule of PCCs in the acute neurosurgical setting should be investigated in further studies.

As expected, coagulation function improved after 4-PCC administration in all patients. Similarly to our findings, a retrospective study of 26 patients (35% of whom had intracranial hemorrhage) reported that the mean INR decreased from 5.7 ± 6.1 (range from 1.6 to 30) to 1.5 ± 0.4 (range from 1.2 to 2.6) after 4-PCC administration [29]. In a multicentered study of 103 patients presenting with INR of ≥2.0, the INR decreased to ≤1.3 after 30 min after 4-PCC administration in 62.2% of patients and in 9.6% of patients receiving FFP, indicating that 4-PCC is more effective than FFP for rapid INR reversal. Similarly, another study in patients with INR of ≥2.0 found that INR decreased to ≤1.3 in 93% of patients within 30 min of 4-PCC administration [30,31]. Importantly, INR remained between 1.2 and 1.3 during the 48-h follow-up period, suggesting that 4-PCC is associated with rapid and stable reversal of VKA-associated coagulopathy.

In our series, more than half of patients died during the hospitalization period. Patients who died presented with greater disease severity relative to survivors, while coagulopathy level and 4-PCC dose were similar as a function of in-hospital mortality. Advanced age and greater initial disease severity are associated with poor prognosis of acute neurosurgical patients [32], and VKA use is more common with advancing age. Patients presenting with VKA-associated intracranial bleeding should be considered at greater risk of complications and poor outcomes, despite perioperative coagulopathy reversal. 

Retrospective design and small sample size are the major limitations of the study that prevented us from prospectively recording disease severity, postoperative complications, and timing of INR correction. Moreover, the study sample was small, despite including additional patients since the implementation of the 4-PCC protocol. On the other hand, our cohort included elderly and very sick patients that are commonly found in routine clinical practice, but are usually excluded from clinical trials.

## 5. Conclusions

The 4-PCC facilitates INR reversal and surgery in patients presenting with warfarin-associated coagulopathy and intracranial bleeding, requiring urgent neurosurgical intervention. The mortality rate is high and is associated with greater disease severity among patients presenting with warfarin-associated intracranial bleeding, despite PCC administration. A greater than expected post-operative intracranial re-bleeding rate underscores the need of careful intraoperative hemostasis, and monitoring of coagulation function, after neurosurgical intervention. Prospective studies examining optimal PCC administration protocol(s) in the acute neurosurgical setting are strongly warranted.

## Figures and Tables

**Table 1:** Demographic and clinical characteristics (*n* = 35).

Characteristic	Value
Gender, *n* (%)	
Male	17 (49)
Female	18 (51)
Age, median (range), years	72 (39–87)
History of warfarin use, *n* (%)	35 (100)
History of liver diseases, *n* (%)	1 (3)
Diagnosis, *n* (%)	
Acute subdural hematoma	19 (53)
Chronic subdural hematoma	3 (9)
Supratentorial spontaneous intracerebral hematoma	7 (20)
Cerebellar spontaneous intracerebral hematoma	4 (12)
Aneurysmal subarachnoid hemorrhage	1 (3)
Bleeding into metastatic angiosarcoma	1 (3)
ASA physical status classification system, *n* (%)	
Class III	3 (23)
Class IV	20 (57)
Class V	7 (20)
GCS	
GCS score, median (range)	7 (3–15)
GCS score 3–8, *n* (%)	20 (57)
GCS score 9–12, *n* (%)	10 (29)
GCS score 13–15, *n* (%)	5 (14)
Admission laboratory tests results	
Platelet count, median (range), ×10^9^/L	203.50 (98.00–353.00)
Platelet count ≤100, *n* (%)	2 (6)
Red blood cell count, median (range), ×10^12^/L	4.36 (2.17–5.99)
White blood cell count, median (range), ×10^9^/L	11.4 (4.6)
Urea, median (range), mmol/L	5.6 (3.00–78.00)
Creatinine, median (range), µmol/L	87.50 (55.90–478.00)
K^+^, median (range), mmol/L	4.00 (3.00–5.10)
Na^+^, median (range), mmol/L	137.50 (122.00–157.00)
Glucose, median (range), mmol/L	8.21 (1.03–14.58)
Coagulation correction/transfusion	
Octaplex	
Dose, median (range), I.U.	2000 (500–3000)
Dose 500–1000, *n* (%)	8 (23)
Dose 1000–2000, *n* (%)	18 (51)
Dose 2000–3000, *n* (%)	9 (26)
Fresh frozen plasma transfusion, *n* (%)	1 (3)
Vitamin K, *n* (%)	35 (100)
Platelet transfusion, *n* (%)	1 (3)
Red blood cell transfusion, *n* (%)	4 (11)
Surgical procedures, *n* (%)	
Osteoplastic craniotomy	14 (40)
Decompressive craniectomy	8 (23)
External ventricular drainage	8 (23)
Burr-holes	3 (9)
Intracranial pressure monitoring	1 (3)
Craniotomy and aneurysm clipping	1 (3)

GCS: Glasgow coma scale.

**Table 2:** Coagulation function before and after 4-PCC administration.

Characteristic	Before Operation	After Operation	*Z* Value, *p* Value
INR, median (range)	2.94 (3.52)	1.32 (1.04–2.52)	−4.78, <0.01
INR, *n* (%):			
<1.20	1 (3)		
1.20–2.00	10 (29)	24 (73)	
2.00–2.50	3 (9)	1 (3)	
2.50–3.00	4 (12)	1 (3)	
3.00–3.50	4 (12)	0 (0)	
>3.50	12 (35)	0 (0)	
PT, median (range), %	56 (21–87)	18 (5–67)	−4.64, <0.01
PTT, median (range), s	41 (24.65)	36 (27–49)	−2.40, 0.02

INR: international normalized ratio; PT: prothrombin time; PTT: partial thromboplastin time.

**Table 3:** Complications and outcomes (*n* = 35).

Characteristic	Value
GOS, median (range), score	1 (1–5)
Score 1–3, *n* (%)	24 (69)
Score 4–5, *n* (%)	11 (31)
In-hospital mortality, *n* (%)	
Yes	20 (57)
No	15 (43)
In-hospital complications, *n* (%)	
Any	15 (43)
Pneumonia	9 (26)
Kidney failure	1 (3)
Meningitis	1 (3)
Sepsis	2 (6)
Subdural empyema	1 (3)
Urinary tract infection	1 (3)
Post-PCC thrombosis	0 (0)
Postoperative intracranial bleeding, *n* (%)	
No	31 (88)
Acute subdural hematoma	2 (6)
Acute epidural hematoma	2 (6)

GOS: Glasgow outcome scale; PCC: prothrombin complex concentrate.

**Table 4:** Clinical characteristics as a function of in-hospital mortality.

Characteristic	In-Hospital Mortality		*p* Value
	Died	Survived,	
	(*n* = 20, 57%)	(*n* = 15, 43%)	
Admission GCS, median (range), score	4 (3–12)	12 (4–15)	0.06
Age, median (range), years	74 (43–87)	63.00 (39–78)	0.36
Hospital stay, median (range), days	9 (1–55)	23 (7–87)	0.001
INR before surgery, median (range)	3.21 (1.38–8.60)	2.79 (1.53–8.16)	0.13
INR after surgery, median (range)	1.38 (1.04–2.52)	1.32 (1.06–1.86)	0.56
PLT count before surgery, median (range), ×10^9^/L	222 (98–353)	187 (100–255)	0.23
Octaplex dose, median (range), IU	2000 (1000–3000)	1500 (500–3000)	0.33
Postoperative bleeding, *n* (%)	1 (5)	3 (20)	0.29
Postoperative infection, *n* (%)	7 (35)	7 (47)	0.36

GCS: Glasgow coma scale; INR: international normalized ratio; PLT: platelet.
